# Validation of 3D intraoral scanner use for detecting caries lesions around dental restorations: A diagnostic accuracy study

**DOI:** 10.1007/s00784-026-07226-7

**Published:** 2026-10-19

**Authors:** Bruna Machado Fernandes, Anelise Fernandes Montagner, Sarah Arangurem Karam, Françoise Helene van de Sande, Marcos Britto Correa

**Affiliations:** 1 Graduate Program in Dentistry, Federal University of Pelotas, R. Gonçalves Chaves 457, room 706, Pelotas, 96015-560 Brazil; 2 Graduate Program in Health and Behavior, Catholic University of Pelotas, Pelotas, Brazil

**Keywords:** Secondary caries, Intraoral scanner, CARS criteria, Diagnostic accuracy, Sensitivity, Specificity

## Abstract

**Objectives:**

This study aimed to validate the use of a 3D intraoral scanner (IOS) for detecting secondary caries lesions around restorations using image-based CARS-derived severity categories, compared with visual-tactile clinical examination.

**Materials and methods:**

A diagnostic validation study was conducted at the university clinical facilities, from July 2023 to September 2024, including adult patients with amalgam or composite resin restorations on anterior and posterior teeth. The restorations were assessed using visual-tactile clinical examination, adopted as the clinical reference standard, and IOS image-based assessment. Sensitivity, specificity, predictive values, binary-classification AUC, and clinical–IOS agreement were estimated. Confidence intervals for diagnostic accuracy measures were obtained using participant-level cluster bootstrap resampling.

**Results:**

A total of 299 restorations in 41 patients were assessed. IOS sensitivity varied, with 75% (95% CI: 66.2–82.0) at the initial lesion threshold, 40% (95% CI: 20.8–57.7) at the moderate lesion threshold, and 57.1% (95% CI: 33.3–100.0) at the severe lesion threshold. Specificity was consistently high, with 84.1% (95% CI: 78.7–89.4), 97.4% (95% CI: 95.6–99.2), and 99.7% (95% CI: 98.7–99.7) at the initial, moderate, and severe lesion thresholds, respectively. Binary-classification AUC values were 0.79 (95% CI: 0.75–0.84), 0.69 (95% CI: 0.58–0.78), and 0.78 (95% CI: 0.66–1.00) at the initial, moderate, and severe lesion thresholds, respectively. Positive predictive value was highest at the severe lesion threshold (80%), while the negative predictive value was especially high at this threshold (99%). Exact clinical-IOS agreement was 77.9%, whereas linearly weighted observed agreement was 91.1%, with a linearly weighted kappa coefficient of 0.53.

**Conclusions:**

The 3D intraoral scanner is a complementary tool for assessing secondary caries lesions, showing high specificity across lesion thresholds. Sensitivity varied according to lesion severity and was less precise at the severe threshold due to the small number of severe lesions. Future research should increase sample sizes and explore combining IOS with other diagnostic methods to improve detection, especially for moderate lesions and higher caries prevalence.

**Clinical Relevance:**

Conventional IOS images may assist in documenting and assessing visible changes around restorations. Clinical interpretation should consider the observed sensitivity limitations and incorporate visual-tactile examination and radiographic assessment when indicated.

**Supplementary Information:**

The online version contains supplementary material available at https://doi.org/10.1007/s00784-026-07226-7.

## Introduction

Secondary caries lesions, also referred to as caries lesions around restorations, are among the main reasons for restorative intervention, repair, or replacement, regardless of the restorative material [1]. Secondary caries lesions are defined as “lesions at the margins of existing restorations” or “lesions associated with restorations or sealants” [2]. These types of lesions develop around an existing dental restoration and are associated with marginal failures in the restoration, allowing bacteria to penetrate and initiate the demineralization process of the dental structure [3]. This process is influenced by factors such as inadequate oral hygiene, high frequency of cariogenic food consumption, and the characteristics of the restorative material and technique used. Alongside tooth and restorations fractures, secondary caries lesions are the leading cause of restoration replacement [4]. If left undiagnosed and untreated, these lesions may progress and lead to further damage of the tooth structure [3].

The diagnosis of secondary caries lesions presents a clinical challenge due to the presence of an associated restoration [5, 6]. Currently, clinical examination combined with radiography is considered the conventional diagnostic method for secondary caries [7]. The clinical exam evaluates the integrity of the restoration on visible surfaces, while radiography allows the observation of proximal lesions, assessing their presence, depth, and relationship to the pulp cavity [8]. Specific visual criteria, such as CARS (Caries Associated with Restorations or Sealants), assist in guiding evaluation by focusing on the integrity of restoration margins and the presence of signs of active demineralization [9]. Additionally, CARS has been suggested as a more conservative diagnostic strategy for assessing restorations [10].

Technological advancements in dentistry have introduced new tools, such as the 3D intraoral scanner, initially developed as an alternative to traditional impression techniques [11]. Regarding alternative detection methods, the increased use of 3D intraoral scanners in clinical practice has also opened opportunities for diagnostic applications [12]. This technology captures real-time three-dimensional images, providing complete and precise visualization of dental structures, including clinically challenging angles.

Recent studies have investigated the diagnostic performance of IOS for caries detection, mainly in primary caries lesions. Evidence suggests that IOS may present moderate diagnostic accuracy, with better performance for occlusal and dentin caries lesions, and that sensitivity and specificity tend to improve as lesion severity increases [13]. In an in vitro study of occlusal surfaces of extracted human teeth, assessment using 3D-coloured IOS images showed substantial agreement with direct visual examination for ICDAS classification, although initial lesions were more frequently misclassified [14].

On-screen assessment of 3D models has also shown performance comparable to visual clinical examination for occlusal caries detection, supporting its potential use in screening, remote assessment, and epidemiological settings [15, 16]. Also, has been reported that using the fluorescence-based caries detection feature of a TRIOS 4 scanner, there’s a higher diagnostic accuracy for moderate-to-advanced lesions than for initial lesions. However, correlation between automated scanner classifications and visual assessment was weak on restored occlusal surfaces [17]. In addition, IOS systems incorporating near-infrared imaging or transillumination have demonstrated promising results for proximal and interproximal caries detection [12, 18], while automated approaches using artificial intelligence applied to 3D scans have also shown encouraging diagnostic performance [19]. However, available evidence is still heterogeneous [20], and most studies have focused on primary caries in unrestored teeth or on scanners with additional optical caries-detection technologies. Therefore, the performance of IOS for detecting caries lesions around restorations remains insufficiently explored. To our knowledge, no previous clinical validation study has investigated the diagnostic performance of conventional 3D intraoral scanner images for detecting caries lesions around restorations according to the CARS criteria.

Based on this, this study aimed to validate the use of a 3D IOS for detecting secondary caries lesions around restorations using image-based CARS-derived severity categories, compared with visual-tactile clinical examination. The hypothesis was that IOS images would show diagnostic accuracy comparable to visual-tactile clinical examination for detecting caries lesions around restorations, with performance varying according to lesion severity.

## Materials and methods

### Study design

The present study was designed as a clinical validation study of diagnostic accuracy to assess the performance of 3D intraoral scanner images as an index test for detecting caries lesions around restorations using image-based CARS-derived severity categories. Visual-tactile clinical examination was adopted as the reference standard for comparison with IOS based assessment. In this study, the term clinical reference standard refers to the best available clinical comparator for in vivo diagnosis of caries lesions around restorations, based on trained examiner judgment and standardized CARS criteria. The study was conducted at the dental clinic of the School of Dentistry, Federal University of Pelotas (UFPel), Brazil. Data collection took place from July 2023 to September 2024.

This study was reported following the STAndard Reporting of CARies Detection and Diagnostic Studies (STAR CARDDS) guideline [21] (Supplementary file, Table S1).

### Ethical aspects

Ethical approval was granted by the Research Ethics Committee of the Federal University of Pelotas (UFPEL) (number 1.625.236/2016). The procedures complied with the ethical standards of the institutional and national research committee, as well as the 1964 Helsinki Declaration and its subsequent amendments. All participants signed an informed consent form

This diagnostic accuracy validation study was not registered in a clinical trial database because it was not an interventional clinical trial; participants were not allocated to treatment groups, and no therapeutic or preventive intervention was tested.

### Participants

Regular patients attending the dental clinic of the School of Dentistry, Federal University of Pelotas (UFPel), were invited to participate. Initially, patients were asked about the presence of restorations, and after an initial evaluation by a researcher involved in the study, those meeting the eligibility criteria were invited to participate.

#### Inclusion criteria

Participants were eligible if they were 18 years old or older and had at least one permanent anterior or posterior tooth with an existing amalgam or composite resin restoration. No restriction was applied regarding restoration surface involvement or extent.

#### Exclusion criteria

Temporary restorations were excluded. No exclusion criterion was applied based on restoration extent or surface involvement, provided that the restored tooth could be clinically examined and adequately captured by the intraoral scanner.

### Sample size

Sample size calculation considered the following parameters: a 20% prevalence of secondary caries lesions, sensitivity of 85%, specificity of 85%, a precision of 10% points, and a 95% confidence interval. Based on these data, a total of 245 restorations were required for the study. The estimated prevalence of secondary caries was based on the review by Demarco et al. (2023), which reported restoration survival rates ranging from 43% to 99% depending on the study characteristics and observation period. Considering that the present study was conducted in a university clinical setting and included restorations with varying service times, most of them between 5 and 10 years, we assumed an overall survival rate of approximately 80%. Consequently, a prevalence of 20% of failed restorations due to caries was considered.

### Training and calibration of evaluators

Three examiners performed the assessments (two performed the clinical assessment and one performed the 3D scanner images assessment). Before evaluations, the examiners underwent training and calibration procedures. Calibration was conducted in three phases. In the first phase, the scanner examiner was trained in equipment handling under the supervision of an experienced professional who demonstrated proper device use, allowing the examiner to practice scanning techniques. In the second phase, the two examiners provided theoretical training, presenting the image examiner with clinical photographs of restored teeth with various CARS scores for case discussion. In the third phase, a sequence of 3D scanned images not included in this study was projected for all three examiners, who, positioned separately, recorded their diagnoses individually based on CARS criteria.

Interexaminer reproducibility for the image examiner was assessed using percentage agreement and weighted kappa, with the two experienced clinical examiners as references. The image examiner showed 94.8% agreement with the first reference examiner, with a weighted kappa of 0.80. Agreement with the second reference examiner was 90.1%, with a weighted kappa of 0.75.This training and calibration process was specifically conducted to standardize the examiner responsible for IOS image assessment before the analysis of the study images.

### Clinical protocol

#### Diagnosis criteria

To conduct the study, the visual CARS (Caries Associated with Restorations or Sealants) criterion was established as the assessment parameter. This criterion, described by the ICCMS (International Caries Classification and Management System) [22], considers the presence of caries around restorations based on clinical characteristics. Based on clinical appearance and the assigned score (ranging from 0 to 6), management recommendations vary according to lesion severity and activity; scores ≥ 4 generally indicate operative intervention, although treatment decisions should also consider patient caries risk and complementary clinical or radiographic findings when indicated.

#### Clinical evaluation

After dental prophylaxis, clinical evaluations were performed by two trained and calibrated examiners using a dental mirror and a ball-point probe under operatory light, with air drying of the dental surfaces. Clinical evaluations were performed independently by two trained and calibrated examiners. Each restoration was clinically assessed by a single examiner, and duplicate examinations of the same restoration by both examiners were not performed. The visual-tactile assessment of restorations strictly followed the established criteria for diagnosing caries lesions around restorations, specifically using the Caries Associated with Restorations and Sealants (CARS) criteria. Both examiners had prior experience with clinical studies employing this method.

The clinical reference standard was established through visual-tactile examination performed by trained and calibrated examiners under standardized clinical conditions, including dental prophylaxis, air drying, operatory light, dental mirror, and ball-point probe. Replicate examinations were not performed for each patient. Therefore, the clinical reference standard should be interpreted as an expertise-based clinical standard, supported by examiner training, calibration, standardized diagnostic criteria, and direct clinical assessment [23]. The restoration, rather than the individual surface or margin, was the unit of assessment. Each restoration received a single CARS score corresponding to the highest code observed at any clinically accessible margin. Separate surface-level scores were not recorded, and the restoration was retained as the unit of analysis.

Patients were seated in a dental chair, and the visual-tactile examination—recognized as the conventional approach for caries detection—was carried out accordingly. Evaluations were guided by a pre-recorded list of restored teeth, and the corresponding CARS scores were meticulously documented by an assistant on standardized forms, using coded participant identifiers.

The visual-tactile examinations were conducted after the intraoral scanning procedures. Upon completion, all records were organized and securely stored for subsequent analysis.

#### Scanner evaluation

Intraoral scans were performed using the TRIOS 3^®^ 3D scanner (3Shape TRIOS A/S, Copenhagen, Denmark), supported by TRIOS software version 1.18.2.11 and Dental Desktop version 1.6.8.1 (3Shape TRIOS A/S, Copenhagen, Denmark). All teeth were scanned under standard white light to produce 3D models accurately reflecting the natural tooth color, with teeth kept moist throughout the procedure. Images were saved in DCM and STL formats and exported for further analysis. Scans were considered adequate when the software captured sufficient detail of the examined teeth, strictly following the manufacturer’s guidelines.

One week after each data collection, the calibrated examiner analyzed the 3D images on a high-resolution screen using 3Shape 3D Viewer software (3Shape TRIOS A/S, Copenhagen, Denmark) and assigned image-based CARS-derived severity categories based on the visual information available on the 3D models. For each restoration, the highest severity category identified across the margins visible on the 3D model was recorded as the restoration-level IOS classification. Replicate on-screen assessments of the IOS images were not performed; therefore, intraexaminer reproducibility for IOS assessment was not estimated. For IOS assessment, the CARS criteria were operationalized based on the visual information available on the 3D models. Accordingly, the examiner considered visible changes in color, opacity, marginal discoloration, surface discontinuity, shadowing, and cavitation adjacent to restorations. Criteria that require dynamic clinical conditions, such as evaluation after air-drying, observation of wet surfaces, or tactile confirmation with a dental probe, could not be directly reproduced during on-screen assessment. To reduce overinterpretation of individual CARS codes in this context, IOS findings were grouped into lesion severity categories: sound, initial, moderate, and severe lesions. Diagnostic performance was then analyzed using cumulative disease severity thresholds.

**Fig. 1:**
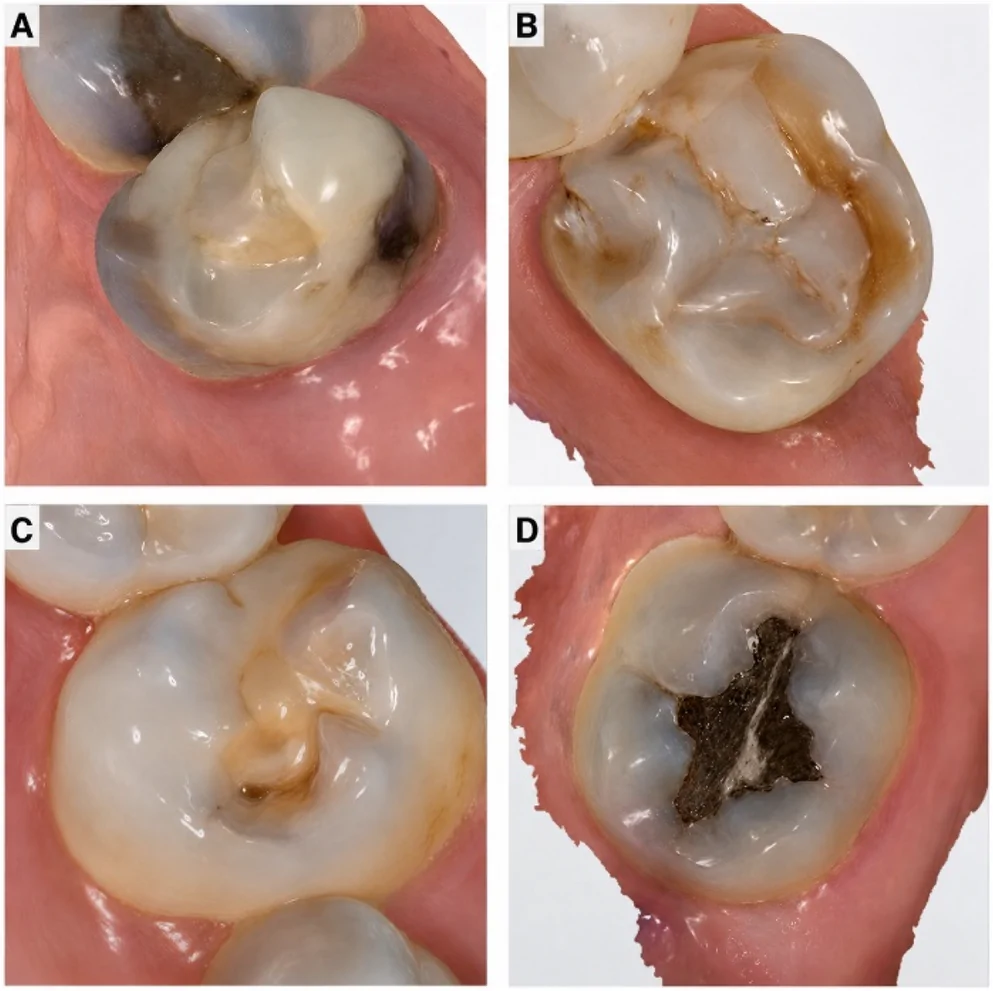
Representative intraoral scanner images of restored posterior teeth (**A**–**D**)

Once the 3D image diagnoses were completed, results were entered into an Excel database using coded participant identifiers and tooth numbers. Concurrently, an assistant recorded all clinical exam results in a separate Excel sheet, including only clinical data. The scanner examiner was not involved in the clinical assessments and was blinded regarding the clinical evaluation scores. Figure 1 shows representative intraoral scanner images of restored posterior teeth.

### Data analysis

The Stata 18.0 software (StataCorp, College Station, TX, USA) was used for data analysis. Contingency tables were constructed for clinical outcomes, comparing diagnoses obtained from intraoral scanner images with those from clinical examination (reference standard).

Agreement between clinical and intraoral scanner assessments across the four ordinal diagnostic categories (0–3) was evaluated using exact percentage agreement, linearly weighted observed agreement, and Cohen’s linearly weighted kappa coefficient. Linear weights were applied using the kap command with the wgt(w) option.

Diagnostic accuracy was assessed at the restoration level, using clinical examination as the reference standard. Sensitivity, specificity, area under the receiver operating characteristic curve (AUC), positive and negative likelihood ratios, and positive and negative predictive values were calculated. To account for the correlation between multiple restorations within the same participant, 95% confidence intervals for all diagnostic accuracy measures were estimated using participant-level cluster bootstrap resampling with 2,000 replications. Analyses were performed using the bootstrap command with the cluster(pct) option, where pct identified individual participants. Percentile-based 95% confidence intervals were obtained using the estat bootstrap, percentile postestimation command. The same procedure was consistently applied across all diagnostic thresholds. As the IOS classifications were binary, the area under the receiver operating characteristic curve (AUC) was calculated as (sensitivity + specificity)/2, corresponding to balanced accuracy. Thus, the reported AUC values represent the performance of binary diagnostic classifications rather than a multi-threshold ROC analysis.

For parameter calculation, outcomes were dichotomized according to each lesion severity threshold, with restoration as the unit of analysis. Diagnostic accuracy parameters were calculated using cumulative cut-off points based on lesion severity. For the initial lesion threshold, CARS code 0 was classified as absence of secondary caries, whereas CARS codes 1–6 were classified as presence of secondary caries lesions. For the moderate lesion threshold, CARS codes 0–2 were classified as absence of moderate or severe secondary caries lesions, whereas CARS codes 3–6 were classified as presence of moderate or severe secondary caries lesions. For the severe lesion threshold, CARS codes 0–4 were classified as absence of severe secondary caries lesions, whereas CARS codes 5–6 were classified as presence of severe secondary caries lesions. Therefore, at each threshold, lesions classified in more advanced CARS categories were included in the disease group.

## Results

A total of 59 individuals were invited to participate in the study. After an initial examination, 18 did not meet inclusion criteria due to absence of direct restorations. There were no refusals. A total of 299 restorations were evaluated across 41 patients, of which 46 restorations (15.4%) were located in anterior teeth and 253 in posterior teeth (84.6%). The sample consisted of patients aged between 18 and 60, comprising 34 women and 7 men. The mean number of restorations per person included was 7.29, ranging from 2 to 17.

Table 1 shows the frequencies of caries lesions around restorations classified using the clinical CARS assessment and the IOS image-based CARS-derived severity categories, categorized as sound, initial, moderate, and severe. According to the clinical evaluation, 75.9% of restorations were classified as sound, while 15.7%, 6.0%, and 2.3% were diagnosed as initial, moderate, and severe lesions, respectively. In the IOS evaluation, 69.9% of restorations were classified as sound, with initial, moderate, and severe lesion prevalence rates of 24.4%, 4.0%, and 1.7%, respectively.

**Table 1:** Frequency of restoration diagnosis according to CARS visual criteria codes

Lesion stage	Clinical – *N* (%)	IOS – *N* (%)
Sound	227 (75.9)	209 (69.9)
Initial	47 (15.7)	73 (24.4)
Moderate	18 (6.0)	12 (4.0)
Severe	7 (2.3)	5 (1.7)

Table 2 presents the clinical–IOS intermethod agreement and reliability for caries lesions around restorations based on the CARS visual criteria. A linearly weighted agreement of 91.1% was observed, with a weighted kappa index of 0.53, indicating moderate agreement between the two diagnostic methods.

**Table 2:** Clinical–IOS intermethod agreement and reliability based on CARS based on CARS-derived severity categories (*N* = 299)

Clinical diagnosis	IOS diagnosis	IOS diagnosis	IOS diagnosis	IOS diagnosis	IOS diagnosis
	Sound	Initial	Moderate	Severe	Total
Sound	191	29	6	1	227
Initial	13	34	0	0	47
Moderate	5	9	4	0	18
Severe	0	1	2	4	7
Total	209	73	12	5	299
Exact clinical–IOS agreement = 77.9% Linearly weighted observed agreement = 91.1% Weighted Kappa = 0.528					

Exact agreement represents the proportion of restorations assigned to identical categories by both diagnostic methods. Linearly weighted observed agreement accounts for the magnitude of disagreement across the four ordinal diagnostic categories (0–3), using weights defined as (w_{ij} = 1 - |i-j|/3). Cohen’s weighted kappa was calculated using the same linear weighting scheme

Table 3 presents the diagnostic properties of the intraoral scanner for detecting caries lesions around restorations according to cumulative CARS disease severity thresholds. Sensitivity was 75% (95% CI: 66.2–82.0) at the initial lesion threshold, 40% (95% CI: 20.8–57.7) at the moderate lesion threshold, and 57.1% (95% CI: 33.33–100.0) at the severe lesion threshold. Specificity was 84.1% (95% CI: 78.7–89.4), 97.4% (95% CI: 95.6–99.2), and 99.7% (95% CI: 98.7–99.7) at the initial, moderate, and severe lesion thresholds, respectively. The area under the ROC curve was 0.79 (95% CI: 0.75–0.84) at the initial lesion threshold, 0.69 (95% CI: 0.58–0.78) at the moderate lesion threshold, and 0.78 (95% CI: 0.66–1.00) at the severe lesion threshold, indicating higher diagnostic performance at the initial and severe thresholds than at the moderate threshold.

**Table 3:** Diagnostic properties of the scanner for detecting caries lesions around restorations according to cumulative CARS disease severity thresholds (*N* = 299)

	Severity of decay (merged CARS)	Severity of decay (merged CARS)	Severity of decay (merged CARS)
Parameter	Initial lesion threshold	Moderate lesion threshold	Severe lesion threshold
Sensitivity (%)	75.0 (66.2; 82.0)	40.0 (20.8; 57.7)	57.1 (33.3; 100.0)
Specificity (%)	84.1 (78.7; 89.4)	97.4 (95.6; 99.2)	99.7 (98.7; 99.7)
AUC	0.79 (0.75; 0.84)	0.69 (0.58; 0.78)	0.78 (0.66; 1.00)
LR+	4.73 (3.57; 6.96)	15.7 (6.20; 50.9)	166.8 (36.3; 260.0)
LR-	0.29 (0.21; 0.39)	0.61 (0.43; 0.82)	0.43 (0.0; 0.67)
PPV (%)	60.0 (47.0; 73.2)	58.8 (29.7; 84.6)	80.0 (33.3; 88.8)
NPV (%)	91.4 (88.0; 94.4)	94.7 (91.6; 97.2)	99.0 (97.7; 1.00)
Kappa	0.54	0.43	0.66

Positive likelihood ratios were higher at more severe thresholds, with values of 4.73 (95% CI: 3.57–6.96) at the initial lesion threshold, 15.7 (95% CI: 6.20–50.9) at the moderate lesion threshold, and 167.0 (95% CI: 36.3–260.0) at the severe lesion threshold. Negative likelihood ratios were 0.29 (95% CI: 0.21–0.39), 0.61 (95% CI: 0.43–0.82), and 0.43 (95% CI: 0.0–0.67) at the initial, moderate, and severe lesion thresholds, respectively. Positive predictive values were 60%, 58.8%, and 80% at the initial, moderate, and severe lesion thresholds, respectively, with the highest value observed at the severe lesion threshold. At the severe lesion threshold, NPV was 99.0%, and the clinical prevalence of severe lesions was 2.3% (7/299). IOS classified four of these seven restorations as severe and assigned lower severity categories to the remaining three. Threshold-specific kappa coefficients for agreement between clinical and IOS classifications ranged from 0.43 to 0.66.

## Discussion

Although some studies have investigated the use of 3D intraoral scanners for diagnosing primary caries lesions [17, 24, 25], to the best of our knowledge, this is the first study to explore the clinical viability of intraoral scanners for detecting caries lesions around restorations using the CARS visual criteria. Overall, the IOS demonstrated satisfactory diagnostic capability in detecting secondary caries lesions across initial, moderate, and severe stages.

The use of visual-tactile clinical examination as the clinical reference standard should be understood within the clinical context of secondary caries diagnosis. Caries lesions around restorations are commonly assessed through direct clinical judgment supported by standardized visual criteria, and the CARS system provides a structured clinical framework for classifying these lesions according to severity. In diagnostic accuracy studies in which an expertise-based clinical judgment is used as the comparator, the reference standard may be imperfect, and its uncertainty should be acknowledged when interpreting accuracy estimates [23]. Therefore, in the present study, visual-tactile clinical examination was adopted as the clinical reference standard, while recognizing that this standard does not eliminate diagnostic uncertainty.

In this study, the main diagnostic properties studied were sensitivity, specificity, area under the ROC curve, positive predictive value, negative predictive value, and reproducibility (kappa). Sensitivity reflects the ability to accurately identify caries lesions with scanner images, being highest at the initial stage, lowest for moderate lesions, and moderate for severe lesions. This indicates good accuracy in early lesion detection, challenges with moderate lesions, and reasonable capability in detecting severe lesions. Specificity, measuring accuracy in detecting sound surfaces, was high at all stages, suggesting a low probability of false positives. The area under the ROC curve reflects the overall accuracy with scanner images, with good performance in initial and severe lesions and limited performance in moderate lesions. Positive (PPV) and negative predictive values (NPV) indicate diagnostic confidence, particularly for ruling out severe lesions given the high NPV. Finally, kappa values, which measure interexaminer agreement, ranged from moderate to good, with lower values for moderate lesions, possibly indicating variability in lesion interpretation at this stage.

It is worth noting that disease prevalence directly impacts the interpretation of diagnostic parameters, especially for sensitivity and specificity. Disease prevalence refers to the proportion of individuals in a population with the condition at a specific time [26]. Higher prevalence of the outcome generally increases study sensitivity, as more disease cases facilitate true positive cases identification. Conversely, specificity may be compromised, as a lower number of healthy individuals increases the chance of false positives [27]. In this study, secondary caries prevalence in restorations directly influenced results, with the best sensitivity values observed using initial lesions as the cut-off, where prevalence was higher [26].

Additionally, understanding the disease spectrum is essential to analyze how different disease stages affect diagnostic test accuracy [28]. Disease spectrum refers to the variability in severity and clinical presentation of a condition, from initial to advanced stages. Generally, advanced stage cut-off points tend to have higher sensitivity due to greater clarity in diagnosis. Accordingly, greater sensitivity would be expected using severe lesions as the cut-off. However, severe lesion prevalence was quite low, which likely influenced the findings. We believe that a higher number of severe lesions would result in higher sensitivity values, as clearer lesion presentation typically yields better performance. Moderate lesion performance, however, may be limited regardless of prevalence, as intermediate stage lesions have subtler characteristics that are more challenging to detect through isolated imaging [29].

Moderate lesions indeed present a significant challenge, as they include small enamel cavities and dentin shadowing. Small cavities are detectable in visual-tactile examination with a ball-point probe, however, in imaging-based diagnoses, where detection is visual only, some of these lesions may go undetected, which is reflected in the IOS’s lower sensitivity for such lesions. Adding radiographic imaging can improve sensitivity by revealing the depth of cavitated lesions, which is especially useful for advanced stages of caries and for hard-to-visualize areas, such as proximal lesions [29].

However, radiography may also lead to overdiagnosis [30], which is undesirable in restoration analysis. In initial lesion detection, combining radiography with 3D imaging is considered unnecessary, as the IOS already provides good accuracy at this stage. Radiographs are limited in accurately identifying early caries development, which may lead to misdiagnosis or delayed detection [30, 31]. Furthermore, minor restoration imperfections, such as wear or bonding changes between material and dental structure, may be misinterpreted as secondary caries on radiographs, leading to overdiagnosis. This could result in unnecessary interventions, such as premature restoration replacement or invasive treatments in areas unlikely to exhibit significant lesion progression [3, 29].

For moderate lesions, the IOS faces limitations in identifying them, and radiographs may be useful in resolving uncertainties and ensuring a more accurate diagnosis, especially for lesions affecting the inner half of the dentin that require restorative interventions. For severe lesions, combining 3D and radiographic images is advantageous, as it allows a comprehensive view of the tooth, showing lesion extent and depth for effective diagnosis using both resources. Therefore, further studies should explore the viability of diagnosing caries lesions around restorations by combining IOS images with radiographs.

Currently, studies on caries lesions diagnosis largely use ICDAS criteria, widely accepted for this purpose [32]. However, specific diagnostic criteria, such as FDI (International Dental Federation) [33] and CARS (Caries Associated with Restorations and Sealants), exist for identifying lesions around restorations. The FDI criteria are based on three spheres—aesthetic, functional, and biological properties. These categories have 16 subcategories; each scored from 1 to 5 for restorations [33]. The FDI classifies restorations based on parameters such as marginal defects, marginal discoloration, and wear, often leading to early restorative intervention. Considering marginal defects and changes as lesion indicators, FDI may result in overdiagnosis and overtreatment [34]. Meanwhile, CARS, derived from the ICDAS (International Caries Detection and Assessment System), created by the ICCMS (International Caries Classification and Management System), was recently updated to CariesCare 4D [22, 35]. CARS uses the same classification system as ICDAS, with a 0–6 scale, where 0 indicates a sound surface, and 6 characterizes a cavitated surface with exposed dentin, involving more than 50% of the surface. Each surface also receives an assessment of lesion activity. While FDI tends to be more interventionist due to its focus on marginal defects, CARS adopts a more conservative approach, avoiding treatments in cases where lesion progression is unclear [36]. In this study, CARS was chosen for its proven stability in intervention decisions, focusing on a less invasive approach. Future studies should examine the impact of FDI criteria on secondary caries lesion diagnosis using 3D scanners, comparing intervention rates and clinical outcomes with CARS.

Although CARS was used in the present study, it is important to recognize that this criterion was originally developed for direct clinical examination. Therefore, its use on IOS images should be interpreted as an image-based adaptation rather than a direct reproduction of the clinical assessment. Some CARS features can be evaluated on 3D color models, such as marginal discoloration, visible opacity, surface discontinuity, shadowing, and cavitation adjacent to restorations. However, other features, such as the response to air-drying, wet-surface evaluation, and tactile confirmation with a probe, cannot be reproduced on static IOS images. For this reason, future studies should develop and validate an IOS-specific version of CARS, defining which clinical features are reliable on 3D scans and whether grouped severity thresholds are more appropriate than individual CARS codes for image-based assessment.

In addition, future studies should evaluate whether IOS systems with adjunct optical caries-detection features provide additional diagnostic value for caries lesions around restorations. Recent evidence using fluorescence-based imaging in restored teeth also supports the potential value of adjunctive optical methods for secondary caries detection, while reinforcing that imaging-derived findings should not be used as the sole basis for treatment decisions [37]. These technologies may improve detection of proximal lesions or dentin involvement, but they may also increase false positive findings due to restorative materials, marginal staining, gaps, or optical artifacts. Therefore, their performance should be validated separately from conventional color 3D scanning before being incorporated into IOS based criteria for secondary caries lesions detection.

This study’s findings have significant practical applications in dentistry, underscoring the potential of IOS in detecting secondary caries lesions, with several clinical advantages. From a clinical perspective, the main advantage of IOS based assessment appears to be its ability to support the longitudinal evaluation of restored teeth under standardized visual documentation. This may be particularly relevant in follow-up appointments, when clinicians need to distinguish between stable marginal changes and progressive caries related alterations around restorations. In this context, IOS images may assist in monitoring visible marginal discoloration, opacity, surface discontinuity, and cavitation over time, especially in patients with multiple restorations or increased caries risk. The high specificity observed in the present study also suggests that IOS may be useful as a complementary tool to support conservative management when no evident lesion is detected on accessible surfaces. However, this advantage is mainly applicable to clinically visible areas, whereas proximal surfaces, cervical/subgingival margins, and suspected dentin involvement still require careful clinical judgment and, when indicated, radiographic assessment, consistent with current consensus recommendations that consider visual examination the primary diagnostic method and radiography and other technologies as adjuncts [38]. Another notable advantage is the ability to share digital images for remote analysis, promoting collaboration with other centers, especially in the context of multicenter clinical studies on restoration longevity, standardizing secondary caries evaluation as an outcome.

Overall, this study’s results showed better diagnostic performance when initial and severe lesion thresholds were considered. Although sensitivity varied according to lesion severity, specificity remained high across thresholds, and the positive predictive value was highest for severe lesions. Notably, these lesions are of greatest concern for clinicians, as their diagnosis often necessitates invasive procedures, such as repair or complete restoration replacement. Thus, IOS based assessment may be particularly useful as a complementary tool in the evaluation of restored teeth, especially when combined with clinical judgment and, when indicated, adjunct radiographic examination.

It is important to note some study limitations. As discussed above, the use of CARS on IOS images represents an image-based adaptation rather than a direct reproduction of the clinical assessment. This may have affected the detection of lesions with subtle clinical features, particularly at the moderate lesion threshold, which may require tactile assessment or controlled wet/dry visualization for more precise classification. As a result, the sensitivity of IOS at the moderate lesion threshold may have been underestimated.

Another methodological limitation is that replicate on-screen assessments of the IOS images were not performed. Therefore, although interexaminer reproducibility was assessed during the calibration process, intraexaminer reproducibility for IOS assessment could not be estimated. This may have introduced some degree of variability in scan interpretation, particularly for subtle lesions. In addition, the clinical reference standard itself may have underestimated caries lesions around restorations in areas with limited visibility, especially proximal surfaces or regions beneath the restoration-tooth interface. In these cases, IOS findings not detected during visual-tactile examination may have been classified as false positives, potentially affecting specificity and positive predictive values. Additionally, the surface extent of restorations was not systematically recorded. Thus, all restorations affecting different surfaces were assessed collectively. This should be considered when interpreting the findings, since IOS diagnostic performance may vary according to restoration location, surface extension, and clinical visibility.

Another important limitation of IOS based assessment is the potential difficulty in differentiating caries-related findings from marginal defects, surface irregularities, discoloration, shadowing, or scanning artefacts. These features may resemble caries-associated changes on digital models, especially around restoration margins, and may have contributed to false-positive findings. Although predefined CARS criteria and examiner calibration were used to reduce subjective interpretation, marginal defects and scanning artefacts were not recorded as separate diagnostic categories. Therefore, their specific contribution to misclassification could not be directly quantified in this study.

The uncertainty of the diagnostic estimates should also be considered. Although high specificity was observed across thresholds, some estimates presented wide 95% confidence intervals, especially at the severe lesion threshold. This imprecision is likely related to the low prevalence of severe lesions in the sample, which reduces the precision of sensitivity and likelihood ratio estimates. Therefore, results for severe lesions should be interpreted cautiously and confirmed in studies with a larger number of restorations presenting advanced CARS scores.

Furthermore, the high cost of IOS may limit its applicability in some clinical settings. Another limitation of this study was the absence of radiographs as part of the reference standard. Although visual-tactile clinical examination is widely used for detecting caries lesions around restorations and allowed direct assessment according to the CARS criteria, radiographic examination has been reported as a useful adjunct to clinical examination for detecting secondary caries lesions and is particularly relevant for proximal surfaces and lesions extending into dentin [7]. Therefore, lesions located in areas with limited direct visibility, particularly proximal surfaces, may not have been fully captured by the visual-tactile reference standard alone [7, 38]. In addition, anterior and posterior restorations, as well as amalgam and composite resin restorations, were analyzed collectively because the study was not designed or powered to provide reliable stratified diagnostic accuracy estimates according to tooth region or restorative material. Future studies should explore the combined use of IOS and radiographs and evaluate diagnostic performance across different lesion thresholds, restoration locations, restorative materials, and surface involvement.

## Conclusion

The findings of this study support the use of conventional color-textured 3D intraoral scanner images as a complementary method for assessing caries lesions around restorations using image-based CARS-derived severity categories. Specificity remained high across lesion thresholds, whereas sensitivity varied according to lesion severity and was less precisely estimated at the severe threshold due to the small number of severe cases. IOS may contribute to the assessment and documentation of restored teeth, but its findings should be interpreted together with complete clinical examination and radiographic assessment when indicated.

Future studies should include larger numbers of moderate and severe lesions and investigate the combined use of IOS with other diagnostic methods to improve diagnostic performance across lesion stages.

## Supplementary Information

Below is the link to the electronic supplementary material.

Supplementary Material 1

## Data Availability

The data collected are available upon reasonable request, subject to authorization by the authors.

## References

[1] Opdam NJM, Bronkhorst EM, Loomans BAC, Huysmans MCDNJM (2014) Longevity of posterior composite restorations: a systematic review and meta-analysis. J Dent Res 93:943–949. 10.1177/0022034514544217 PMC4293707 25048250

[2] Mjör IA, Toffenetti F (2000) Secondary caries: a literature review with case reports. Quintessence Int 31:165–179 11203922

[3] Askar H, Krois J, Gostemeyer G et al (2020) Secondary caries: what is it, and how it can be controlled, detected, and managed? Clin Oral Investig 24:1869–1876. 10.1007/s00784-020-03268-7 32300980

[4] Demarco FF, Cenci MS, Montagner AF et al (2023) Longevity of composite restorations is definitely not only about materials. Dent Mater 39:1–12. 10.1016/j.dental.2022.11.009 36494241

[5] Seemann R, Flury S, Pfefferkorn F, Lussi A, Noack MJ (2014) Restorative dentistry and restorative materials over the next 20 years: a Delphi survey. Dent Mater 30(4):442–448. 10.1016/j.dental.2014.01.013 Epub 2014 Feb 9. PMID: 24522149 24522149

[6] Nedeljkovic I, De Munck J, Vanloy A et al (2020) Secondary caries: prevalence, characteristics, and approach. Clin Oral Investig 24:683–691. 10.1007/s00784-019-02894-0 31123872

[7] Brouwer F, Askar H, Paris S, Schwendicke F (2016) Detecting secondary caries lesions: a systematic review and meta-analysis. J Dent Res 95:143–151. 10.1177/0022034515611041 26464398

[8] Silva EL, Silva Filho JN, Santos JFF et al (2021) Cárie dentária: considerações clínicas e radiográficas para seu diagnóstico. Salusvita 40:70–88

[9] Machiulskiene V, Campus G, Carvalho JC et al (2020) Terminology of dental caries and dental caries management: consensus report of a workshop organized by ORCA and Cariology Research Group of IADR. Caries Res 54:7–14. 10.1159/000503309 31590168

[10] Digmayer Romero VH, Signori C, Uehara JLS et al (2024) Diagnostic strategies for restorations management: a 70-month randomized clinical trial. J Dent Res 103:697–704. 10.1177/00220345241247773 38752325

[11] Kihara H, Hatakeyama W, Komine F et al (2020) Accuracy and practicality of intraoral scanner in dentistry: a literature review. J Prosthodont Res 64:109–113. 10.1016/j.jpor.2019.07.010 31474576

[12] Michou S, Vannahme C, Bakhshandeh A, Ekstrand KR, Benetti AR (2022) Intraoral scanner featuring transillumination for proximal caries detection: an in vitro validation study on permanent posterior teeth. J Dent 116:103841. 10.1016/j.jdent.2021.103841 34624420

[13] Rashid F, Farook TH, Dudley J (2025) Comparison of the accuracy of intraoral scanners, intraoral cameras, radiographs, and histological methods for the diagnosis of dental caries: a systematic review and meta-analysis. BDJ Open 11:82. 10.1038/s41405-025-00345-5 PMC12518662 41083454

[14] Valdivia Tapia AC, Lara JS, Martinez-Mier EA et al (2025) 3D intraoral scanner as an auxiliary tool for the diagnosis of dental hard-tissue conditions. Clin Oral Investig 29:605. 10.1007/s00784-025-06683-w PMC12678547 41345325

[15] Daneris AP, Karam SA, Mendes FM, Cenci MS, Demarco FF, Correa MB (2025) Validation of intraoral scanner as a tool for the epidemiological diagnosis of caries. J Dent 160:105913 10.1016/j.jdent.2025.105913 40541916

[16] Ntovas P, Michou S, Benetti AR et al (2023) Occlusal caries detection on 3D models obtained with an intraoral scanner: a validation study. J Dent 131:104457. 10.1016/j.jdent.2023.104457 36858167

[17] Agius AM, No Cortes J, Cortes ARG, Attard NJ, Gatt G (2025) Diagnostic performance of an intraoral scanner for caries detection on permanent tooth surfaces. Caries Res. 10.1159/000549789 41343418

[18] Kanar Ö, Tagtekin D, Korkut B (2024) Accuracy of an intraoral scanner with near-infrared imaging feature in detection of interproximal caries of permanent teeth: An in vivo validation. J Esthet Restor Dent 36(6):845–857. 10.1111/jerd.13198 38263949

[19] Ahmed WM, Azhari AA, Alnakhli R, Alsulami Y, Merdad Y, Abdelrazek M, Sahlol AT (2025) Development and evaluation of an artificial intelligence (AI) model for detecting dental caries from 3D intraoral scans. J Prosthet Dent 134:1115e1. 1115.e8 10.1016/j.prosdent.2025.06.020 40664593

[20] Schlenz MA, Schupp B, Schmidt A, Wöstmann B, Baresel I, Krämer N, Schulz-Weidner N (2022) New Caries Diagnostic Tools in Intraoral Scanners: A Comparative In Vitro Study to Established Methods in Permanent and Primary Teeth. Sensors 22:2156. 10.3390/s22062156 PMC8950989 35336328

[21] Neuhaus KW, Eggmann F, Kühnisch J et al (2022) STAndard reporting of caries detection and diagnostic studies (STARCARDDS). Clin Oral Investig 26:1947–1955. 10.1007/s00784-021-04173-3 PMC8816754 34623505

[22] Pitts NB, Ekstrand K (2013) International caries detection and assessment system (ICDAS) and its international caries classification and management system (ICCMS): methods for staging of the caries process and enabling dentists to manage caries. Community Dent Oral Epidemiol 41:41–52. 10.1111/cdoe.12025 24916677

[23] Walsh T (2018) Fuzzy gold standards: Approaches to handling an imperfect reference standard. J Dent 74(1):S47–S49. 10.1016/j.jdent.2018.04.022 29929589

[24] Lin WS, Alfaraj A, Lippert F, Yang CC (2023) Performance of the caries diagnosis feature of intraoral scanners and near-infrared imaging technology: a narrative review. J Prosthodont 32:114–124. 10.1111/jopr.13770 37701946

[25] Litzenburger F, Heck K, Kaisarly D et al (2022) Diagnostic validity of early proximal caries detection using near-infrared imaging technology in 3D range data. Clin Oral Investig 26:543–553. 10.1007/s00784-021-04032-1 PMC8791888 34636940

[26] Li J, Fine JP (2011) Assessing the dependence of sensitivity and specificity on prevalence in meta-analysis. Biostatistics 12:710–722. 10.1093/biostatistics/kxr008 PMC4042906 21525421

[27] Leeflang MM, Bossuyt PM, Irwig L (2009) Diagnostic test accuracy may vary with prevalence: implications for evidence-based diagnosis. J Clin Epidemiol 62:5–12. 10.1016/j.jclinepi.2008.04.007 18778913

[28] Murad MH, Lin L, Chu H et al (2023) The association of sensitivity and specificity with disease prevalence: analysis of 6909 studies of diagnostic test accuracy. CMAJ 195:E925–E931. 10.1503/cmaj.221802 PMC10356012 37460126

[29] Abdelaziz M (2023) Detection, diagnosis, and monitoring of early caries: the future of individualized dental care. Diagnostics 13:3649. 10.3390/diagnostics13243649 PMC10742918 38132233

[30] Kapor S, Rankovic MJ, Khazaei Y et al (2021) Systematic review and meta-analysis of diagnostic methods for occlusal surface caries. Clin Oral Investig 25:4801–4815. 10.1007/s00784-021-04024-1 PMC8342337 34128130

[31] Gimenez T, Tedesco TK, Janoian F et al (2021) What is the most accurate method for detecting caries lesions? A systematic review. Community Dent Oral Epidemiol 49:216–224. 10.1111/cdoe.12641 33847007

[32] Sá G, Michou S, Bönecker M et al (2024) Diagnostic validity of ICDAS clinical criteria on digital 3D models. J Dent 149:105274. 10.1016/j.jdent.2024.105274 39084547

[33] Hickel R, Peschke A, Tyas M et al (2010) FDI World Dental Federation: clinical criteria for the evaluation of direct and indirect restorations—update and clinical examples. Clin Oral Investig 14:349–366. 10.1007/s00784-010-0432-8 20628774

[34] Signori C, Uehara JLS et al (2022) Comparison of two clinical approaches based on visual criteria for secondary caries assessments and treatment decisions in permanent posterior teeth. BMC Oral Health 22:77. 10.1186/s12903-022-02112-6 PMC8931988 35300657

[35] Martignon S, Pitts NB, Goffin G et al (2019) CariesCare practice guide: consensus on evidence into practice. Br Dent J 227:353–362. 10.1038/s41415-019-0678-8 31520031

[36] Moro BLP, Freitas RD, Pontes LRA et al (2020) Influence of different clinical criteria on the decision to replace restorations in primary teeth. J Dent 101:103421. 10.1016/j.jdent.2020.103421 32615237

[37] Bittar A, Alkan E, Tağtekin D (2026) Diagnostic accuracy of QrayPen fluorescence scoring for secondary caries in repair-/replacement-indicated posterior composite restorations. Clin Oral Investig 30:342. 10.1007/s00784-026-07038-9 42467381

[38] Kühnisch J, Aps JKM, Splieth C et al (2024) ORCA-EFCD consensus report on clinical recommendation for caries diagnosis. Paper I: caries lesion detection and depth assessment. Clin Oral Investig 28:227. 10.1007/s00784-024-05597-3 PMC10957694 38514502

