# Supplementary material for: Validation of 3D intraoral scanner use for detecting caries lesions around dental restorations: A diagnostic accuracy study
Source: Clin Oral Investig. 2026 Oct 19;30(11):538. doi: 10.1007/s00784-026-07226-7 (PMC13677634; doi:10.1007/s00784-026-07226-7)
Supplement: Supplementary file 1 [file 784_2026_7226_MOESM1_ESM.docx]

Supplementary file
Table S1. Checklist of the STAndard Reporting of CAries Detection and Diagnostic Studies (STAR CAR DDS)

| **Section and Topic** | **Item No.** | **STARD Recommendations** | **Page** |
| --- | --- | --- | --- |
| **Title** | 1 | Identification as a study of diagnostic accuracy using at least one measure of accuracy (such as sensitivity, specificity,  predictive values, or AUC) | p. 1 |
| **Abstract** | 2 | Structured summary of study design, methods, results, and conclusions | pp. 2-3 |
| **Introduction** |  | Introduce & justify |  |
| Background/rationale | 3 | Scientific and clinical background, including the intended use and clinical role of the  index test | p. 3-4 |
| Objectives | 4 | Study objectives and hypotheses | p. 2, 4 |
| **Methods** | | | |
| Study design | 5 | Whether data collection was planned before the index test and reference standard were performed (prospective study) or after  (retrospective study) | p.4 |
| Participants | 6 | Eligibility criteria | p.5 |
|  | 7 | On what basis potentially eligible participants were identified (such as symptoms, results from previous tests,  inclusion in registry) | p.5 |
|  | 8 | Where and when potentially eligible  participants were identified (setting, location and dates) | pp. 4-5 |
|  | 9 | Whether participants formed a consecutive, random or convenience series | - |
| Test methods | 10a | Index test, in sufficient detail to allow replication | pp. 7-8 |
|  | 10b | Reference standard, in sufficient detail to allow replication | pp. 6-7 |
|  | 11 | Rationale for choosing the reference standard (if alternatives exist) | pp. 4, 6–7;p. 10 |
| Study material/ population | 12a | Definition of and rationale for test positivity cut-offs or result categories of the index test,  distinguishing pre-specified from exploratory | pp. 7–8 |
|  | 12b | Definition of and rationale for test positivity cut-offs or result categories of the reference standard, distinguishing pre-specified from exploratory | pp. 6, 8 |
|  | 13a | Whether clinical information and reference standard results were available to the performers/readers of the index test | pp. 7–8 |
|  | 13b | Whether clinical information and index test results were available to the assessors of the reference standard | pp. 7–8 |
|  | 14 | Methods for estimating or comparing measures of diagnostic accuracy | p. 8 |
|  | 15 | How indeterminate index test or reference standard results were handled | - |
|  | 16 | How missing data on the index test and reference standard were handled | - |
|  | 17 | Any analyses of variability in diagnostic  accuracy, distinguishing pre-specified from exploratory | - |
|  | 18 | Intended sample size and how it was determined | p. 5 |
| **Results** | | | |
| Population and/or teeth | 19 | Flow of participants, using a diagram | - |
| Descriptive | 20 | Baseline demographic and clinical characteristics of participants | p. 8 |
| Outcome data | 21a | Distribution of severity of disease in those with the target condition | p. 21 |
|  | 21b | Distribution of alternative diagnoses in those without the target condition | p. 21 |
|  | 22 | Time interval and any clinical interventions between index test and reference standard | pp. 7–8 |
|  | 23 | Cross tabulation of the index test results (or their distribution) by the results of the reference standard | p. 22 |
|  | 24 | Estimates of diagnostic accuracy and their precision (such as 95% confidence intervals) | p. 23 |
|  | 25 | Any adverse events from performing the index test or the reference standard | - |
| **Discussion** | | | |
| Key results | 26 | Study limitations, including sources of potential bias, statistical uncertainty, and  generalizability | pp. 13–14 |
| Interpretation | 27 | Implications for practice, including the  intended use and clinical role of the index test | pp. 13–14 |
| **Other information** | | | |
| Conflicts of interest | 28 | Registration number and name of registry | p.5 |
|  | 29 | Where the full study protocol can be accessed | - |
|  | 30 | Sources of funding and other support; role of funders | p. 1 |
